# REIMAGINING AGING: EXAMINING THE IMPACT OF LIFETIME DISCRIMINATION EXPERIENCES ON EVERYDAY METAMEMORY

**DOI:** 10.1093/geroni/igac059.2959

**Published:** 2022-12-20

**Authors:** Timothy Ly, Jeanne Cundiff, Jason DeCaro, Rebecca Allen

**Affiliations:** The University of Alabama, Tuscaloosa, Alabama, United States; The University of Alabama, Tuscaloosa, Alabama, United States; The University of Alabama, Tuscaloosa, Alabama, United States; The University of Alabama, Tuscaloosa, Alabama, United States

## Abstract

Lifetime and recent experiences of discrimination (based on race, gender, religion, sexual orientation, etc.) contribute to impaired performance on cognitive assessments. However, the underlying mechanism by which discrimination negatively impacts cognition is unclear. Recent research investigating stress-induced impairment of metamemory may address the relationship between discrimination experiences and cognitive impairment. The aim of this study was to determine the relationship between lifetime experiences of discrimination, especially recent experiences, and everyday metamemory from a lifespan perspective (ages 20–75), using data collected from the Midlife in the United States (MIDUS Refresher 1) Daily Diary Project (N = 782). Results from a 2-level multilevel model showed that the relationship between recent experiences of discrimination explained unique variance in impaired metamemory accuracy (25%; β = .377, σ = .052, 95% CI [.275, .479]), suggesting that individuals with recent discrimination experiences reported more cognitive complaints. Furthermore, the relationship between age, recent experiences of discrimination, and impaired metamemory showed that younger individuals reported more complaints after experiencing discrimination than older individuals (β = .192, σ = .092, 95% CI [.011, .373]. Individual differences accounted for 45% of the variance in the number of cognitive complaints. These findings demonstrate the need for more research into understanding metamemory accuracy as an underlying mechanism by which the psychosocial stressor of discrimination impacts cognition across the lifespan. Moreover, understanding the experiences of diverse aging populations, including experiences of discrimination, and their impact on cognition will inform research on interventions to promote positive cognitive health outcomes across the lifespan.

